# ELMO1 and Rac1 Signaling in Kidney Disease: Molecular Mechanisms, Context-Dependent Roles, and Therapeutic Potential

**DOI:** 10.3390/biomedicines14081660

**Published:** 2026-07-23

**Authors:** Licheng Xie, Wenyao Jia, Xitong Xu, Huijuan Wu

**Affiliations:** 1 Department of Pathology of School of Basic Medical Sciences, Fudan University, Shanghai 200032, China; xielicheng0127@163.com (L.X.);; 2 Medical School of Nantong University, Nantong 226001, China

**Keywords:** engulfment and cell motility 1 (ELMO1), diabetic kidney disease (DKD), IgA nephropathy (IgAN), focal segmental glomerulosclerosis (FSGS), acute kidney injury (AKI), renal fibrosis, Rac1, chronic kidney disease (CKD)

## Abstract

**Background:** Chronic kidney disease (CKD) is a major global health burden, affecting more than 850 million people worldwide. Increasing evidence implicates engulfment and cell motility 1 (ELMO1), a cytoplasmic adaptor protein that regulates cytoskeletal remodeling, phagocytosis, and immune responses through the ELMO1–DOCK1–Rac1 signaling axis, in renal injury and disease progression. **Methods:** This narrative review identified relevant publications through searches of PubMed, Web of Science, and Google Scholar using combinations of the terms “ELMO1,” “kidney disease,” “diabetic kidney disease,” “IgA nephropathy,” “focal segmental glomerulosclerosis,” “acute kidney injury,” and “renal fibrosis.” English-language original research articles, genetic association studies, mechanistic investigations, and selected review articles were preferentially included according to their relevance to ELMO1 in kidney diseases. **Results:** Genetic association studies have implicated the ELMO1 locus in susceptibility to diabetic kidney disease (DKD), although associated variants and effect sizes differ across populations. Experimental studies suggest that ELMO1 regulates cytoskeletal remodeling, inflammatory responses, oxidative stress, and extracellular matrix deposition through the canonical ELMO1–DOCK1–Rac1 signaling pathway as well as Rac1-independent mechanisms. Available evidence supports a role for ELMO1 in DKD and renal fibrotic remodeling. In focal segmental glomerulosclerosis, the relevance of ELMO1 is primarily inferred from Rac1-associated podocyte injury pathways, whereas evidence in acute kidney injury remains limited and context-dependent. ELMO1 may contribute to inflammatory injury in ischemia–reperfusion settings but may also support efferocytosis and tissue repair in nephrotoxic injury models. In IgA nephropathy, evidence for a direct role of ELMO1 remains limited and is currently based largely on indirect mechanistic observations involving mucosal immunity and glomerular injury responses. **Conclusions:** ELMO1 is a context-dependent regulator of cytoskeletal, inflammatory, oxidative, and matrix-remodeling processes relevant to kidney disease. The strongest evidence currently supports its involvement in DKD, whereas its roles in other kidney diseases remain to be further defined in disease-specific, cell-type-specific, and stage-specific experimental models. Further studies are required to clarify its potential as a biomarker or therapeutic target in CKD.

## 1. Introduction

Chronic kidney disease (CKD) affects more than 850 million individuals worldwide and poses a substantial global health burden [1]. According to the Global Burden of Disease study, CKD is among the leading causes of mortality, reflecting its increasing prevalence and clinical impact [2]. Glomerular diseases, including diabetic kidney disease (DKD), IgA nephropathy (IgAN), and focal segmental glomerulosclerosis (FSGS), constitute major causes of kidney failure, while acute kidney injury (AKI) and progressive renal fibrosis critically contribute to disease progression and long-term outcomes.

Engulfment and cell motility 1 (ELMO1) encodes a cytoplasmic adaptor protein that forms a bipartite guanine nucleotide exchange factor (GEF) complex with DOCK1 (Dock180), thereby activating the small GTPase Rac1 to regulate actin cytoskeleton dynamics essential for phagocytosis, cell migration, and tissue remodeling [3,4]. Mechanistically, ELMO1 functions as a key signaling adaptor linking cell-surface receptors, including phosphatidylserine receptors involved in apoptotic cell clearance, to Rac1-dependent cytoskeletal reorganization [5,6]. This signaling axis is evolutionarily conserved from Caenorhabditis elegans (CED-12) to mammals, highlighting its fundamental role in innate immune and cellular homeostatic processes [4].

ELMO1 is expressed in multiple cell types and participates in diverse biological processes, including immune regulation, renal homeostasis, and vascular remodeling [7,8,9,10,11]. This functional distribution is consistent with its involvement in cytoskeletal remodeling, phagocytic clearance, and tissue repair.

**Figure 1:**
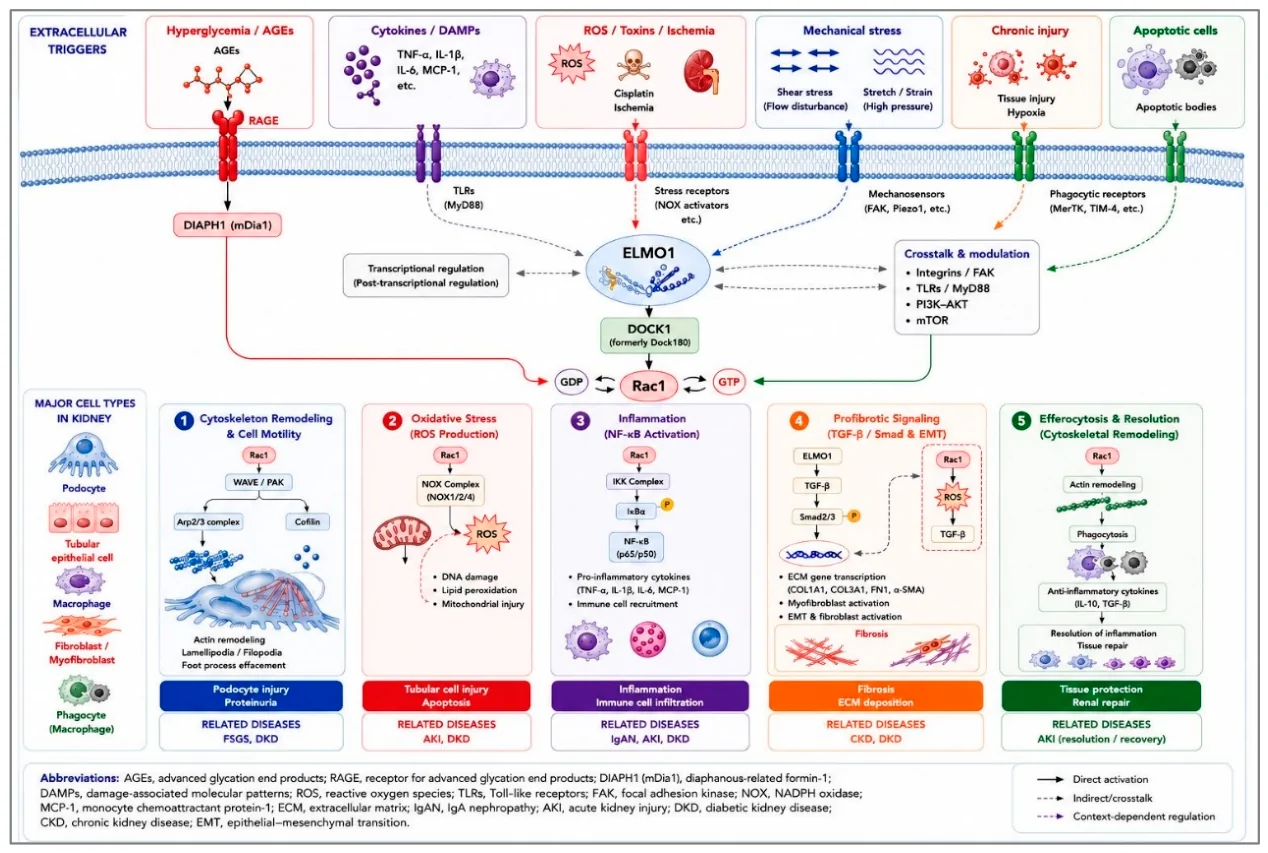
Overview of ELMO1-mediated pathogenesis across kidney diseases.

In kidney disease, ELMO1 was initially identified as a susceptibility gene for DKD through genetic association studies [12], and subsequent studies have confirmed its genetic association across multiple populations [13]. These findings suggest that ELMO1 is involved in renal disease susceptibility. However, available evidence indicates that the biological effects of ELMO1 are highly context-dependent, not only across disease categories but also across experimental systems and injury models. For example, in DKD, sustained metabolic stress has been associated with ELMO1-related amplification of inflammatory signaling, oxidative stress, and extracellular matrix accumulation, contributing to progressive fibrotic remodeling, whereas in acute injury models, including ischemia–reperfusion and toxin-induced injury, ELMO1 has been implicated in phagocytic and efferocytotic pathways that may facilitate injury resolution, depending on the balance between inflammatory activation and reparative responses [14,15]. This apparent functional duality highlights the complexity of ELMO1-mediated signaling in the kidney and suggests that its biological effects are tightly regulated by disease context and cell type. However, the precise regulatory mechanisms underlying these context-specific effects remain incompletely understood. An overview of the proposed context-dependent roles of ELMO1 across major kidney diseases is provided in Figure 1, and the available evidence regarding disease settings, experimental models, relevant cell types, and reported functional effects is summarized in Table 1.

Therefore, this review summarizes current knowledge regarding ELMO1 in kidney diseases, with a focus on its roles in glomerular diseases, AKI, and renal fibrogenesis, and discusses its potential as a context-dependent regulator and therapeutic target in CKD progression.

## 2. Physiological Role of ELMO1 in Innate Immunity and Tissue Homeostasis

Physiologically, ELMO1 coordinates cell motility and phagocytic clearance, processes that are integral to innate immunity and tissue homeostasis. As a core component of the ELMO1–DOCK1–Rac1 signaling axis, ELMO1 functions as a scaffold protein that facilitates Rac1 activation, thereby promoting actin cytoskeletal remodeling necessary for phagocytosis and directed cell migration [3]. Through this pathway, phagocytes recognize and internalize apoptotic cells, preventing secondary necrosis and fostering the release of anti-inflammatory cytokines that contribute to immune tolerance and tissue repair [4,6]. ELMO1 also plays a pivotal role in leukocyte chemotaxis and inflammatory responses. Experimental studies have demonstrated that genetic deletion of ELMO1 impairs neutrophil chemotaxis and adhesion in response to inflammatory stimuli, highlighting its essential role in immune cell trafficking and host defense [37,38]. These findings underscore the importance of ELMO1 in orchestrating innate immune responses during infection and tissue injury. Beyond immunity, ELMO1 contributes to tissue homeostasis and organ development [7,8]. It regulates endothelial cell migration during angiogenesis and participates in maintaining vascular integrity through Rac1-mediated cytoskeletal dynamics [7,8,39]. In the nervous system, ELMO1 is involved in axon guidance, neuronal migration, and synaptic pruning, reflecting its evolutionarily conserved role in cellular remodeling [5,40].

Pathological dysregulation of ELMO1 has been observed in conditions characterized by aberrant Rac1-dependent migration and engulfment. Overexpression of ELMO1 has been reported in several malignancies, where it promotes lamellipodia formation, extracellular matrix degradation, and invasive phenotypes in a Rac1-dependent manner [41,42,43]. Conversely, reduced ELMO1 expression or loss-of-function mutations impairs phagocytic efficiency, leading to the accumulation of apoptotic debris and the persistence of chronic inflammation [4,6]. Functional modulation of ELMO1 also influences downstream signaling pathways involved in cell survival, migration, and extracellular matrix remodeling, contributing to oncogenic or fibrotic cascades [15,44]. Genetic association studies have identified polymorphisms in the *ELMO1* locus as risk factors for metabolic and inflammatory diseases. Notably, intronic variants of *ELMO1* are reproducibly associated with susceptibility to DKD and albuminuria across diverse populations [12,13]. Meta-analyses further confirm that *ELMO1* polymorphisms confer a statistically significant increase in DKD risk [45], implicating this gene in renal fibrogenesis during chronic kidney injury [16].

Emerging evidence also links aberrant ELMO1 expression with cancer progression. Functional studies have shown that ELMO1 overexpression enhances migratory and invasive phenotypes in several tumor cell models, whereas ELMO1 silencing attenuates these effects, supporting a dosage-sensitive role for ELMO1 in tumor cell motility and invasion [41,42,43,44,46].

Clinically, elevated ELMO1 expression is associated with advanced tumor stage and unfavorable clinical outcomes, underscoring its potential as a prognostic biomarker and therapeutic target [44,46].

## 3. Role of ELMO1 in the Pathogenesis of Glomerular Disease

### 3.1. ELMO1 in the Pathogenesis of DKD

The role of ELMO1 in DKD is supported by converging evidence from genetic association studies, functional investigations, and transcriptomic analyses, making it one of the best-characterized pathogenic regulators implicated in diabetic renal injury. Genome-wide association and replication studies have implicated the ELMO1 locus in susceptibility to DKD across several populations, although the associated variants, risk alleles, and effect sizes vary by ancestry. In a Chinese cohort of patients with type 2 diabetes, rs741301 was associated with diabetic nephropathy, and carriage of the A allele remained independently associated with disease after multivariable adjustment [47]. In contrast, an Iranian study associated the G allele and GG genotype of rs741301 with DKD [48], illustrating that the apparent risk allele may differ across populations. In the European-ancestry GoKinD cohort of individuals with type 1 diabetes, the strongest ELMO1 association signals were observed for rs11769038 and rs1882080 rather than rs741301 [13]. Consistent with this heterogeneity, a 2024 meta-analysis of 17 studies found that rs741301 was associated with DKD under homozygous and recessive genetic models, but not under the allelic model [45]. Collectively, these data support ELMO1 as a replicated candidate susceptibility locus for DKD while indicating substantial allelic heterogeneity and ancestry-dependent effects, rather than a uniform association of rs741301 across populations. The current evidence supporting the involvement of ELMO1 in DKD is summarized in Figure 1 and Table 1.

In DKD, ELMO1 may act as a context-dependent molecular link between the diabetic injury milieu and progressive glomerular damage. Persistent hyperglycemia, oxidative stress, advanced glycation end products (AGEs), altered intrarenal hemodynamics, and injury-associated damage-associated molecular patterns (DAMPs) release together establish a pro-oxidative and pro-inflammatory renal microenvironment. Although ELMO1 expression was reported to be elevated in cells cultured under high-glucose conditions [15,17], direct evidence that AGEs, oxidative stress, hemodynamic stress, or DAMPs regulate ELMO1 expression in specific renal cell populations remains limited. Functional studies nevertheless support a role for ELMO1 in renal injury responses. In Akita diabetic mice, reduced Elmo1 expression attenuated albuminuria, glomerular injury, oxidative stress, and profibrotic gene expression, whereas increased Elmo1 expression aggravated these phenotypes, supporting a causal and dosage-dependent relationship between Elmo1 expression and nephropathy severity [16]. In cultured human mesangial cells, ELMO1 promoted fibronectin expression and extracellular matrix dysregulation, including through a Rac1-independent interaction with cyclooxygenase-2 (COX-2); however, these experiments were not performed in fully diabetic settings [17]. Conversely, in hyperglycemic zebrafish, Elmo1 deficiency impaired glomerular architecture and ultrafiltration, while observations in human diabetic kidney samples suggested a potential role for ELMO1 in maintaining glomerular structural integrity under certain conditions [9]. These apparently divergent findings indicate that the biological effect of ELMO1 is determined by expression level, genetic background, cell type, disease stage, and the balance between homeostatic and sustained injury-associated signaling.

In addition, the ELMO1–DOCK1–Rac1 axis provides a biologically relevant framework for interpreting additional processes in DKD. ELMO1–DOCK1 directly activates Rac1, and activated Rac1 regulates actin dynamics through the WAVE regulatory complex, p21-activated kinases (PAKs), and the Arp2/3 complex, promoting branched actin polymerization and lamellipodia formation [49,50]. In podocytes, excessive or sustained Rac1 activation disrupts slit diaphragm integrity and promotes foot process effacement, thereby contributing to proteinuria [51,52,53]. Enhanced Rac1 activity promotes NADPH oxidase (NOX)-mediated reactive oxygen species (ROS) production, which contributes to mitochondrial dysfunction and cellular injury. Excess ROS further amplifies NF-κB–dependent inflammatory signaling, thereby facilitating cytokine production and macrophage recruitment [54,55,56,57]. In parallel, sustained Rac1 signaling may reinforce profibrotic responses through interconnected pathways: Rac1-dependent PI3K/Akt activation can enhance transforming growth factor-β (TGF-β)-induced collagen expression in mesangial cells, whereas oxidative stress and MAPK signaling can potentiate TGF-β/Smad activity in renal injury settings [18,19,20,21]. These convergent pathways favor the expression of α-SMA, fibronectin, and collagen I, thereby promoting extracellular matrix accumulation and renal fibrotic remodeling. Although these Rac1-associated downstream programs are well established in DKD and related renal injury models, the extent to which each is directly mediated by ELMO1 in specific renal cell populations remains incompletely defined. ELMO1 should therefore be viewed as an upstream, context-dependent modulator of Rac1-centered signaling networks rather than as a uniformly pathogenic driver. Its therapeutic relevance requires validation in cell-type-specific and disease-stage-specific DKD models.

### 3.2. The Role of ELMO1 in IgA Nephropathy

Recent large-scale genome-wide association studies have not identified ELMO1 as a susceptibility locus for IgA nephropathy, suggesting that ELMO1 is unlikely to represent a primary genetic determinant of IgAN [22]. Therefore, the potential involvement of ELMO1 in IgAN should currently be interpreted as hypothesis-generating rather than established. The genetic architecture of IgAN did not identify ELMO1 as a susceptibility locus, despite implicating multiple loci related to mucosal immunity, complement activation, and IgA regulation [22]. Therefore, ELMO1 is unlikely to represent a primary germline determinant of IgAN susceptibility. Nevertheless, the absence of a germline genetic association does not exclude disease-related alterations in ELMO1 expression, protein abundance, post-translational regulation, subcellular localization, or cell-type-specific signaling activity during IgAN progression. These possibilities remain unproven and require direct investigation in IgAN tissues and disease-relevant experimental models.

IgAN is increasingly recognized as a mucosal immune-mediated disease in which the gut–kidney axis links intestinal dysbiosis, epithelial barrier dysfunction, persistent host–microbe interactions, and aberrant mucosal immune activation to the generation of galactose-deficient IgA1 (Gd-IgA1), formation of pathogenic circulating immune complexes, and subsequent mesangial deposition in the kidney [58,59,60,61]. Within the intestinal mucosa, ELMO1 has established functions in epithelial cells and macrophages during host–microbe interactions. Bacterial recognition through brain angiogenesis inhibitor 1 (BAI1) recruits the ELMO1–DOCK1 complex and activates Rac1-dependent cytoskeletal remodeling, facilitating engulfment of Gram-negative bacteria [6,10]. During enteric infection, ELMO1 also contributes to autophagy-associated bacterial clearance; ELMO1 deficiency delays bacterial elimination and is associated with enhanced intestinal inflammatory responses [11]. In addition, ELMO1-dependent host–microbe responses can influence innate immune signaling and inflammatory responses [23]. These findings support the hypothesis that altered ELMO1 activity may shape the mucosal inflammatory microenvironment in which abnormal IgA responses arise. However, no IgAN-specific study has demonstrated that ELMO1 directly regulates APRIL or BAFF expression, Gd-IgA1 production, IgA glycosylation, or intestinal barrier integrity.

Following the formation and renal deposition of pathogenic IgA-containing immune complexes, ELMO1 could theoretically influence downstream injury responses in a cell-specific manner. IgAN-associated immune complexes primarily activate mesangial cells, initiating inflammatory and matrix-associated responses that reshape the glomerular microenvironment and contribute to secondary podocyte and tubulointerstitial injury [24,25,26]. Although ELMO1 has not been shown to determine immune-complex deposition, its established roles in mesangial matrix regulation provide a rationale for investigating whether it modifies glomerular remodeling after immune-complex-induced activation. This possibility is supported indirectly by non-IgAN studies showing that ELMO1 regulates fibronectin expression and extracellular matrix production in mesangial cells [15,17]. Inflammatory arthritis and peritonitis models demonstrate that ELMO1 regulates Rac1-dependent adhesion, chemotaxis, and inflammatory-cell recruitment in vivo, with evidence currently derived from neutrophil-mediated inflammatory models [38,62]. These findings suggest that ELMO1 may influence immune-cell behavior within inflamed tissues; however, whether it regulates the recruitment, activation, or inflammatory functions of renal-infiltrating macrophages in IgAN remains unknown. In podocytes, no direct evidence currently links ELMO1 to IgAN-associated injury. Nevertheless, because ELMO1 is an upstream regulator of Rac1-dependent cytoskeletal signaling, altered ELMO1 activity could potentially modify podocyte susceptibility to cytoskeletal destabilization under inflammatory or oxidative stress [27,30]. These renal mechanisms remain hypothetical and require direct validation in IgAN tissues and cell-type-specific experimental models.

Taken together, current evidence supports a well-established role for ELMO1 in regulating host–microbe interactions, mucosal immune responses, and epithelial–macrophage communication within the gut. However, its involvement in IgA nephropathy remains indirect and hypothesis-driven. Although ELMO1 is not supported by genetic association studies and has not been directly investigated in IgAN tissues or disease models, the emerging concept of the gut–kidney axis in IgAN provides a biologically plausible framework in which ELMO1 may contribute to disease progression. This represents an important gap in knowledge and warrants further investigation in future studies.

### 3.3. ELMO1 in the Pathogenesis of FSGS

In FSGS, dysregulated Rac1 signaling in podocytes is increasingly recognized as a central driver of disease pathogenesis. The ELMO1-DOCK1 complex functions as a well-established upstream activator of Rac1, promoting GDP-GTP exchange and sustained Rac1 activation [3,4]. Recent advances using single-cell RNA sequencing (scRNA-seq) and spatial transcriptomics have further refined our understanding of cellular heterogeneity and disease-associated transcriptional states in FSGS and kidney fibrosis. These studies reveal distinct molecular programs associated with podocyte injury, endothelial dysfunction, and fibrogenic remodeling in diseased kidneys [28,29]. Notably, emerging single-cell analyses suggest that podocyte injury is associated with activation of stress-response pathways and dedifferentiation programs, which are closely linked to Rac1-mediated actin remodeling and cell detachment. Clinical biopsy specimens and animal studies further demonstrate enhanced Rac1 pathway activation in podocytes from FSGS patients and models, correlating with foot process effacement and segmental sclerosis [27,30,31]. Given its upstream regulatory role, aberrant upregulation or activation of ELMO1 could potentiate Rac1-driven cytoskeletal instability, rendering podocytes more susceptible to mechanical stress, inflammatory stimuli, and progressive detachment. In addition to cytoskeletal regulation, ELMO1 may contribute to fibrotic remodeling in glomerular disease. Evidence suggests that ELMO1 promotes extracellular matrix production in renal cells through Rac1-dependent signaling pathways [15,17]. Such mechanisms may theoretically contribute to glomerular scarring and segmental sclerosis in FSGS.

Although direct experimental evidence linking ELMO1 specifically to FSGS remains limited, its established role as an upstream activator of Rac1 suggests a plausible contribution to podocyte cytoskeletal instability and disease progression. Supporting this concept, studies in diabetic nephropathy demonstrate that ELMO1 promotes extracellular matrix accumulation, inflammation, and renal injury through Rac1-dependent pathways. Given the shared mechanisms of podocyte injury and glomerulosclerosis between DKD and FSGS, it is reasonable to hypothesize that aberrant ELMO1 activation may similarly exacerbate Rac1-driven podocyte dysfunction in FSGS. The available evidence supporting the involvement of ELMO1-associated signaling in podocyte injury and FSGS is summarized in Figure 1 and Table 1.

## 4. ELMO1 in the Pathogenesis of AKI

Emerging evidence suggests that ELMO1 exerts context-dependent effects in AKI, with distinct roles reported in ischemia–reperfusion injury (IRI) and cisplatin-induced nephrotoxicity. In a bilateral renal IRI model, global ELMO1 deficiency did not significantly alter the immediate decline in renal function, indicating that ELMO1 is not a dominant determinant of the early functional response to acute ischemic injury [14]. However, findings from a separate unilateral IRI model indicate more complex compartment-specific effects. Mice with genetically reduced ELMO1 expression exhibited less severe tubular structural injury but increased urinary albumin excretion after IRI, whereas renal expression of inflammatory mediators, including IL-6, TNF-α, CXCL1, and TLR4, was comparable to that in wild-type mice [32]. These findings suggest that reduced ELMO1 activity may differentially influence tubular injury and glomerular filtration barrier integrity, rather than uniformly suppressing renal inflammation. Consistent with a role in maladaptive repair, genetically increased ELMO1 expression aggravated renal dysfunction, albuminuria, glomerular ultrastructural injury, tubulointerstitial fibrosis, and redox-related transcriptional abnormalities during the late phase of unilateral IRI-induced AKI-to-CKD transition [33].

In contrast, ELMO1 appears to exert a protective role in cisplatin-induced nephrotoxic AKI, a setting characterized by extensive tubular epithelial apoptosis. In this model, global ELMO1 deficiency aggravated renal tissue injury and increased apoptotic-cell accumulation, whereas macrophage-specific ELMO1 deletion did not reproduce the phenotype observed in globally deficient mice [14]. Primary-cell experiments and renal immunofluorescence analyses further suggested that ELMO1 contributes to efferocytosis across both immune and non-immune renal cell compartments [14]. These findings support a model in which ELMO1-dependent apoptotic-cell clearance is distributed across multiple renal cell populations rather than being mediated exclusively by macrophages.

The divergent effects of ELMO1 across AKI models may reflect differences in the dominant injury program, the renal cell populations engaged, and the stage of injury and repair. IRI is characterized primarily by hypoxic–reoxygenation stress, microvascular dysfunction, and inflammatory activation, whereas cisplatin nephrotoxicity is accompanied by extensive tubular epithelial apoptosis. In the latter context, ELMO1 may be particularly important for coordinated apoptotic-cell clearance. Mechanistically, ELMO1 interacts with DOCK1 to activate Rac1-dependent actin remodeling required for phagocytic cup formation and apoptotic-cell internalization [3,4]. In vivo vertebrate studies have shown that ELMO1-deficient macrophages exhibit impaired engulfment of apoptotic cells, providing a mechanistic basis for linking ELMO1 to efferocytosis [34]. Related evidence from ANCA-associated glomerulonephritis indicates that CXCL12/CXCR4-driven downstream Rac1 signaling can enhance macrophage efferocytosis within the kidney; however, its association with crescent formation and fibrosis in this inflammatory setting also highlights that the net consequences of ELMO1-dependent apoptotic-cell clearance are disease-context-dependent [63]. Collectively, current evidence does not support a uniformly pathogenic or protective role for ELMO1 in AKI. Rather, its effects appear to depend on the predominant injury mechanism, the renal cell populations involved, and the temporal phase of injury and repair. Because DOCK1 or Rac1 dependence has not been directly tested in cisplatin-induced AKI, the downstream Rac1 signaling should be regarded as a biologically supported mechanistic framework rather than a fully established causal pathway in nephrotoxic AKI. Future studies using cell-type-specific ELMO1 perturbation, time-resolved analyses, and direct interrogation of DOCK1–Rac1 signaling are needed to define the causal mechanisms underlying these context-dependent effects. The context-dependent effects of ELMO1 across different AKI models, including ischemic and nephrotoxic injury, are summarized in Figure 1 and Table 1.

## 5. Role of ELMO1 in Renal Fibrosis

Initially identified as a susceptibility gene for DKD, ELMO1 has been implicated in promoting ECM accumulation, inflammation, and profibrotic signaling within the kidney [12,15]. Elevated ELMO1 expression has been reported in experimental models of diabetic nephropathy and in renal cells exposed to diabetic stimuli, highlighting its involvement in chronic kidney injury [15,16]. Mechanistically, ELMO1 may contribute to renal fibrogenesis through modulation of Rac1-associated signaling pathways, leading to cytoskeletal remodeling and transcriptional regulation of fibrogenic genes. Activation of Rac1-associated profibrotic pathways has been linked to increased expression of extracellular matrix components, including COL1A1, COL3A1, and FN1, as well as myofibroblast markers such as ACTA2 (α-SMA) and profibrotic mediators including CTGF (CCN2) and TGF-β1, which collectively contribute to epithelial–mesenchymal transition (EMT), fibroblast activation, and ECM accumulation. Rac1 activation promotes ROS generation via NADPH oxidases, which in turn stimulates TGF-β/Smad signaling, a central pathway in renal fibrosis [19,20,54]. These convergent pathways promote excessive ECM deposition and progressive renal fibrosis [21].

In addition to its direct profibrotic effects, ELMO1 may also amplify renal inflammatory responses, which are central drivers of fibrogenesis. Rac1 signaling has been implicated in inflammatory pathway activation, including NF-κB-dependent cytokine production and chemokine-mediated macrophage recruitment, thereby promoting the transcription of pro-inflammatory cytokines, including TNF-α, IL-6, and MCP-1 [35,36]. This cascade enhances monocyte/macrophage recruitment and sustains a persistent inflammatory microenvironment within the injured kidney. Notably, this chronic inflammatory state further potentiates TGF-β–mediated fibroblast activation and ECM deposition, ultimately establishing a feed-forward loop that accelerates tubulointerstitial fibrosis progression [19]. The reported mechanisms linking ELMO1 to renal fibrosis and extracellular matrix remodeling are summarized in Table 1.

## 6. Conclusions and Future Perspectives

ELMO1 is emerging as a context-dependent regulator of kidney disease pathogenesis, integrating cytoskeletal dynamics, inflammatory signaling, oxidative stress, and extracellular matrix remodeling through Rac1-dependent and context-specific pathways. Genetic and functional studies have established a causal role for ELMO1 in DKD, whereas its contribution to FSGS is supported by mechanistic links between ELMO1-associated Rac1 signaling and podocyte injury. In AKI, ELMO1 exhibits distinct and context-dependent effects across injury models, with differential roles in ischemic and nephrotoxic injury, including regulation of inflammatory responses and apoptotic-cell clearance. Moreover, ELMO1 may contribute to renal fibrogenesis by linking cytoskeletal regulation, redox signaling, and profibrotic pathways, suggesting a potential role in chronic kidney disease progression. Although indirect biological links between ELMO1-related immune regulation and IgAN pathogenesis have been proposed, direct disease-specific evidence remains lacking.

From a translational perspective, ELMO1 represents a potential therapeutic and biomarker candidate across kidney diseases. Selective modulation of the ELMO1–Rac1 axis, together with advances in precision medicine, RNA-based therapeutics, and nanoparticle-mediated delivery systems, may provide opportunities for disease-specific intervention. Future studies integrating multi-omics profiling, spatial analyses, and clinically annotated cohorts will be essential to define cell-type-specific and disease-stage-specific functions of ELMO1 and to translate these findings into clinically actionable strategies [64].

## Figures and Tables

**Table 1:** Functional roles of ELMO1 across kidney diseases.

Disease	Major Cell Types	Involved Pathway	Biological Functions	Net Effect	Corresponding References
DKD	Podocytes, tubular epithelial cells, mesangial cells	ELMO1-DOCK–Rac1; TGF-β/Smad; NF-κB	Cytoskeleton remodeling, inflammation, ECM accumulation, oxidative stress	Pathogenic	[9,15,16,17,18,19,20,21]
IgAN	Mesangial cells, macrophages, intestinal epithelial cells	Rac1 signaling; innate immunity pathways	Mesangial proliferation, immune activation, mucosal immune dysregulation	Potentially pathogenic	[10,11,22,23,24,25,26]
FSGS	Podocytes	ELMO1-DOCK-Rac1	Foot process effacement, increased motility, detachment, cytoskeletal instability	Likely pathogenic	[27,28,29,30,31]
AKI (IRI)	Macrophages, tubular epithelial cells, endothelial cells	Rac1; NF-κB; ROS pathways	Inflammatory cell recruitment, oxidative stress, endothelial dysfunction	Injury-promoting Context-dependent	[14,32,33]
AKI (Cisplatin)	Macrophages, tubular epithelial cells	ELMO1–DOCK–Rac1	Efferocytosis, apoptotic cell clearance, inflammation resolution	Protective Context-dependent	[3,4,14,34]
Renal fibrosis	Fibroblasts, tubular epithelial cells	Rac1; TGF-β/Smad; ROS	fibroblast activation, ECM deposition (COL1A1, FN1, α-SMA)	Pro-fibrotic	[18,19,20,21,35,36]

## Data Availability

No new data were created or analyzed in this study.
